# The Clinical Utility of Food Addiction: Characteristics and Psychosocial Impairments in a Treatment-Seeking Sample

**DOI:** 10.3390/nu12113388

**Published:** 2020-11-04

**Authors:** Edgar Oliveira, Hyoun S. Kim, Emilie Lacroix, Mária de Fátima Vasques, Cristiane Ruiz Durante, Daniela Pereira, Janice Rico Cabral, Paula Sanches Bernstein, Ximena Garcia, Emma V. Ritchie, Hermano Tavares

**Affiliations:** 1 Department of Psychiatry, University of São Paulo, Rua Artur de Azevedo, 145, Cerqueira Cesar, São Paulo CEP 05404-010, SP, Brasil; vasquesfatima10@gmail.com (M.d.F.V.); crisnutrition@hotmail.com (C.R.D.); dani.psico8@gmail.com (D.P.); janice.rcabral@gmail.com (J.R.C.); paulabernstein@ciencias.com.br (P.S.B.); hermanot@usp.br (H.T.); 2 Department of Psychology, Ryerson University, 350 Victoria Street, Toronto, ON M5B 2K3, Canada; 3 Department of Psychology, University of Calgary, 2500 University Drive, NW, Calgary, AB T2N 1N4, Canada; emilie.lacroix@ucalgary.ca (E.L.); xgarcia@ucalgary.ca (X.G.); 4 Department of Psychology, York University, 4700 Keele St, Toronto, ON M3J 1P3, Canada; evritchi@yorku.ca

**Keywords:** food addiction, clinical utility, psychosocial impairment, comorbidity, quality of life

## Abstract

Little is known about the characteristics of individuals seeking treatment for food addiction (FA), and the clinical utility of FA has yet to be established. To address these gaps, we examined (i) the demographic, eating pathology, and psychiatric conditions associated with FA and (ii) whether FA is associated with psychosocial impairments when accounting for eating-related and other psychopathology. Forty-six patients seeking treatment for FA completed self-report questionnaires and semi-structured clinical interviews. The majority of the sample were women and self-identified as White, with a mean age of 43 years. Most participants (83.3%) presented with a comorbid psychiatric condition, most commonly anxiety and mood disorders, with a mean of 2.31 comorbid conditions. FA was associated with binge eating severity and anxiety symptoms, as well as psychological, physical, and social impairment. In regression analyses controlling for binge eating severity, food cravings, depression, and anxiety, FA remained a significant predictor only of social impairment. Taken together, the results suggest that individuals seeking treatment for FA are likely to present with significant comorbid conditions, in particular anxiety disorders. The results of the present research provide evidence for the clinical utility of FA, particularly in explaining social impairment.

## 1. Introduction

The food addiction (FA) hypothesis posits that foods that are highly processed and rich in salt, sugar, and fat have “addictive potential”. In other words, certain foods may have the ability to provoke symptoms of substance use disorders [1]. Empirical research on the concept of food addiction is rapidly growing, and animal and human studies have demonstrated similarities between food and drugs that are abused, particularly with respect to their effects on reward pathways in the brain [2,3]. FA, assessed using the self-report Yale Food Addiction Scale [4], which applies substance use disorder criteria to food and eating has been found to be relatively common, ranging from 3% to 20% in non-clinical samples [4]. Rates of FA are higher in eating disorder samples, with some studies finding rates as high as 95% among women with bulimia nervosa [4]. FA is also relatively common in Brazil, with Nunes-Neto and colleagues (2018) reporting a prevalence rate of 4.3% in a large web-based sample [5].

The concept of FA is heavily debated, with some arguing that it pathologizes everyday eating behaviors [6] or does not hold incremental clinical utility beyond existing eating disorder diagnoses [7]. According to the 5th edition of the Diagnostic and Statistical Manual of Mental Disorders, “The diagnosis of a mental disorder should have clinical utility: it should help clinicians to determine prognosis, treatment plans, and potential outcomes for their patients” (p. 20) [8]. However, the clinical utility of FA constitutes an important gap in the field. A systematic review examining the correlates of FA reported a relationship with body mass index, binge eating, and mixed results with other psychopathology including depression [9]. Since the review, studies have also revealed an association between FA and demographic characteristics [10], lower quality of life [11], psychological distress [12,13], problematic eating behaviors [13,14,15], and impulsivity [5,14]. Regarding psychosocial impairments, FA has been shown to have moderate to strong associations that remain significant when other forms of eating pathology are controlled for [16]. Within a Brazilian context, Nunes-Neto and colleagues also found associations between FA and psychopathology and lower quality of life in psychological, physical, social, and environment domains [5]. Thus, there is evidence to suggest that FA may be associated with unique characteristics and psychosocial impairments, providing preliminary support for its clinical utility.

Although these studies are informative, a major limitation is that they have all consisted of non-clinical samples or people experiencing difficulties with weight and eating disorders. Additionally, previous studies have not controlled for the impact of comorbid psychopathology on psychosocial impairments. As such, it is unclear whether the documented clinical characteristics and psychosocial impairments stem from FA or from other conditions, which provides a limited understanding of the clinical utility of FA. Furthermore, the incremental clinical utility of FA is not known, that is, whether it contributes useful information above and beyond what may be explained by the comorbid psychopathology. It is critical to examine the incremental utility of FA among individuals seeking treatment for this concern, given that there is already emerging literature recommending various treatment strategies for FA [17,18]. In this light, the aim of the present study was to address gaps in the understanding of the clinical utility of FA by examining clinical characteristics (demographics, eating disorders, cravings, psychiatric conditions) and psychosocial impairments (controlling for common psychopathology) in a clinical sample with the primary concern of FA.

## 2. Materials and Methods

### 2.1. Participants and Procedures

Participants were 46 patients seeking treatment for food addiction at a specialized university outpatient clinic associated with the University of São Paulo, Faculty of Medicine in São Paulo, Brazil. Patients were self-referred through the community or referred by other treatment programs affiliated with the University. At intake, patients were assessed by a registered psychiatrist to determine their suitability for treatment. The eligibility criteria for treatment were as follows: (i) meet criteria for moderate-to-severe FA as assessed by the Yale Food Addiction Scale 2.0 (YFAS 2.0) [19] and (ii) 18 years of age or over. Exclusion criteria included (i) an organic condition that may explain eating pathology (e.g., endocrine disturbances); (ii) acute psychosis, mania, or borderline personality disorder; (iii) cognitive impairments that may interfere with treatment; and (iv) being currently pregnant or breastfeeding. Current psychiatric conditions were assessed by psychiatrists using the Brazilian Portuguese version of the Mini International Neuropsychiatric Interview (MINI) [20] using the Diagnostic and Statistical Manual of Mental Disorders - IV criteria. The MINI is a brief, semi-structured clinical interview with strong psychometric properties and high inter-rater reliability compared to the Structured Clinical Interview for the DSM [20]. The treatment consisted of 15 weekly sessions of group schema therapy and six sessions of behavioral nutrition delivered by two psychologists and two nutritionists [21].

Ethics approval was obtained from the Faculty of Medicine, University of São Paulo (12820813.5.0000.0068). The research was conducted in accordance with the Declaration of Helsinki. All patients were informed that research participation was voluntary and would not impact their treatment. All participants provided their consent to participate in the study.

### 2.2. Measures

#### 2.2.1. Demographic Information

Participants reported their age, gender, ethnicity, and marital and employment status.

#### 2.2.2. Food Addiction Severity

The Yale Food Addiction Scale 2.0 (YFAS 2.0) [19] was used to assess FA severity. The YFAS 2.0 contains 35 items (α = 0.90) assessing the 11 DSM-5 substance use disorder criteria applied to FA, including two items to assess distress and impairment. The items are anchored from 0 (never) to 7 (every day) with total scores ranging from 0 to 235. In addition to providing a total score, the YFAS 2.0 provides a total symptom count, as well as a categorical “diagnosis” of food addiction (None = ≤1 symptom and/or lack of distress or impairment; Mild = 2 to 3 symptoms plus distress and/or impairment; Moderate = 4 to 5 symptoms plus distress and/or impairment; Severe = ≥6 symptoms plus distress and/or impairment). The YFAS 2.0 was translated and back-translated to Brazilian Portuguese by the senior author (H.T.), a content expert who is fluent in both languages.

#### 2.2.3. Binge Eating Severity

Binge eating severity was assessed using the Brazilian-Portuguese version of the Binge Eating Scale (BES) [22]. The BES consists of 16 items (α = 0.80) assessing the behavioral, affective, and cognitive components associated with binge eating. The response options for BES range from 0 to 3, with two questions containing three response options. As such, the total scores on the BES range from 0 to 46, with higher scores indicating greater binge eating severity.

#### 2.2.4. Food Craving (Trait)

The Brazilian-Portuguese validated version of the Food Cravings Questionnaire—Trait version (FCQ-T) [23] was used to assess the frequency and intensity of participants’ food-related cravings. The FCQ-T contains 39 items (α = 0.94) assessing a variety of dimensions related to food cravings. The items are anchored from 1 (never or not applicable) to 6 (always). Total scores (39–234) are calculated by summing the items with higher scores indicating greater food-related cravings.

#### 2.2.5. Psychiatric Comorbidities

Symptoms of depression and anxiety were assessed using the Brazilian-Portuguese validated versions of the 21-item Beck Depression Inventory (BDI-II) [24] (α = 0.90) and the 21-item Beck Anxiety Inventory (BAI) [25] (α = 0.87), respectively. Both the BDI and BAI are anchored from 0 to 3 with total scores ranging from 0 to 63. The MINI provided diagnostic coverage of current psychiatric conditions, including mood, anxiety, substance use, and eating disorders.

#### 2.2.6. Quality of Life

A Brazilian-Portuguese validated version of the WHOQOL-bref [26] was used to assess quality of life in four domains: psychological, physical, social, and environment. The WHOQOL-bref contains 26 items (α = 0.91) with items ranging from 1 to 5. Mean scores were computed for each domain, with lower scores indicating worse quality of life in the domains.

### 2.3. Data Analytic Plan

First, descriptive statistics were used to examine demographic characteristics and current psychiatric conditions. Next, two-tailed bivariate correlation analyses were conducted to examine associations among YFAS 2.0 total scores, food cravings, binge eating severity, symptoms of depression/anxiety, and quality of life. Lastly, a multiple regression analysis was conducted with the psychological, physical, social, and environmental domains of the WHOQOL-bref as the criterion variable and binge eating severity, food craving, symptoms of depression, and anxiety as the predictor variables to examine whether FA predicted psychosocial impairments above and beyond common comorbidities associated with FA.

## 3. Results

### 3.1. Clinical Characteristics

The sample consisted of more women (*n* = 37; 80.4%) than men (*n* = 9; 19.6%). The mean age was 43.28 (*Standard Deviation [SD]* = 10.82) years. Regarding ethnicity, the majority of the sample self-identified as White (*n* = 36; 78.3%), five as mixed (10.9%), three as Black (6.5%), and one as Asian (2.2%). One participant did not disclose their ethnicity. Roughly half (*n* = 24; 52.2%) of the participants reported being in a relationship, and the majority (*n* = 43; 93.48%) reported being employed. The mean score on the YFAS 2.0 was 9.12 (*SD* = 1.81), which corresponds to severe symptoms of food addiction.

Of the 42 patients who completed the MINI, 35 (83.3%) met criteria for at least one current psychiatric condition, with a mean of 2.31 (*SD* = 1.87) comorbid conditions (Table 1). The most common current psychiatric condition was generalized anxiety disorder (*n* = 26; 61.9%), followed by major depressive episode (*n* = 20; 47.6%), suicidality (*n* = 11; 26.8%), agoraphobia (*n* = 9; 21.4%), and social anxiety (*n* = 8; 19.0%). Regarding eating disorders, seven (16.7%) participants met criteria for bulimia nervosa, and none met criteria for anorexia nervosa. Relatively few, if any, participants met current diagnostic criteria for post-traumatic stress disorder (*n* = 1; 2.4%) or alcohol or substance use/dependence (*n* = 1; 2.4%).

### 3.2. Correlation Analyses

Means, standard deviations, and correlations among the variables of interest are presented in Table 2. Food addiction severity was positively correlated with binge eating severity and anxiety symptoms, and negatively correlated with the psychological, physical, and social domains of the WHOQOL-bref. Food addiction severity was not significantly correlated with food craving, depression symptoms, or the environment domain of the WHOQOL-bref, *p* > 0.055.

### 3.3. Multiple Regression

#### 3.3.1. Psychological Impairment

The multiple regression model predicting psychological impairment was statistically significant F(5,35) = 29.84, *p* < 0.001, adj. R^2^ = 0.78. In this model, only symptoms of depression significantly predicted psychological impairment. FA, binge eating, symptoms of anxiety, and food craving were not significant predictors (Table 3). A post hoc power analysis indicated a power of 1.0 based on our results.

#### 3.3.2. Physical Impairment

The multiple regression model was statistically significant, F(5,35) = 13.36, *p* < 0.001, adj. R^2^ = 0.61. Symptoms of depression and binge eating significantly predicted physical impairments. On the other hand, FA, symptoms of anxiety, and food craving were not significant predictors of physical impairments (Table 4). A post hoc power analysis indicated a power of 0.99 based on our results.

#### 3.3.3. Social Impairment

The multiple regression model was statistically significant, F(5,35) = 4.10, *p* = 0.005, adj. R^2^ = 0.28. FA and symptoms of depression were significant predictors of social impairments. Symptoms of anxiety, binge eating severity, and food craving were not significant predictors (Table 5). A post hoc power analysis indicated a power of 0.95 with the parameters of our observed results. FA symptoms remained a significant predictor of social impairment *b* = −0.049, *t* = −3.20, *p* = 0.004 when controlling for age, gender, marital status, ethnicity, and body mass index.

#### 3.3.4. Environmental

The multiple regression model predicting environmental impairment was statistically significant, F(5,35) = 3.17, *p* = 0.018, adj. R^2^ = 0.21. In this model, only symptoms of depression were significant. FA, binge eating, symptoms of anxiety, and food craving were not significant predictors of environmental impairments (Table 6). A post hoc power analysis indicated a power of 0.88 based on our results.

## 4. Discussion

The present study is, to our knowledge, the first to examine the clinical characteristics and psychosocial impairments of individuals for whom FA is the primary concern and reason for seeking treatment. Regarding demographic characteristics, the sample consisted mainly of women, consistent with previous studies reporting a higher prevalence of FA among women [4]. A potential explanation for this gender difference is that the weight gain associated with or expected to occur as a consequence of food addiction may be more distressing to women given they are under greater sociocultural pressure to conform to a thin body ideal [27]. As a result, women may be more likely to be distressed by and seek treatment for FA. Nonetheless, in the current study, roughly one in five participants seeking treatment were men, providing further evidence for the clinical significance of FA among individuals of both these genders. Future studies investigating gender differences in the clinical characteristics of those seeking treatment for FA would be highly informative. Indeed, to increase the generalizability of characterizations of FA across the gender spectrum, it is important to avoid under-representation of men and individuals who identify as transgender or gender non-binary in studies of FA.

Regarding the relationship between FA and binge eating, the mean score of the participants on the BES was in the severe range, and FA was moderately (*r* = 0.42) associated with binge eating, consistent with previous research [15]. These findings suggest binge eating disorder may be a common comorbidity; however, given that the correlation between FA and BES scores was far from perfect, there does not appear to be a complete overlap between these two constructs. Regarding other eating disorders, no participants met the diagnostic criteria for anorexia nervosa including the binge/purge subtype, and less than 1 in 5 participants met diagnostic criteria for bulimia nervosa. These results may be partly attributable to the low base rates of anorexia nervosa and bulimia nervosa and are consistent with prior evidence that FA can occur outside the context of eating disorders [28,29].

The majority of participants reported a current psychiatric condition, with patients meeting criteria for two diagnoses on average. However, it should be noted that 16.7% of the participants did not meet criteria for any current psychiatric conditions. This result suggests that there likely exists a group of individuals with FA who may not meet criteria for a recognized eating disorder or other psychiatric condition, yet still experience psychosocial impairment severe enough to warrant seeking treatment. Addictive disorders were not very common in the sample, which is surprising given the conceptualization of FA as an addiction and given that addictive disorders tend to co-occur [30,31]. Additionally, FA was not related to cravings, which is also a hallmark symptom of addictive disorders. A potential reason for this finding may be due to the relatively small sample size as the association between FA and craving was approaching significance with close to a moderate effect size (*p* = 0.087, *r* = 0.26). Thus, future research with large samples is needed to further investigate the association of FA with other addictive behaviors, to examine whether they are likely to co-occur, and whether FA is associated with hallmark characteristics of addictive disorders such as heightened levels of impulsivity.

The most frequent comorbid conditions were anxiety and mood disorders. Interestingly, however, FA was not significantly correlated with severity of depression in contrast to previous findings but in line with others [9]. Given the mixed findings in the literature regarding the association between FA and depression, further research is warranted. On the other hand, FA was significantly associated with severity of anxiety. Furthermore, anxiety disorders were the most frequent disorders in patients seeking treatment for FA. Longitudinal studies would be highly valuable in determining the temporal relationships between FA and anxiety.

FA was significantly associated with physical, psychological, and social impairment in our univariate analyses. FA remained a significant predictor of social impairment when controlling for binge eating, craving, depression, and anxiety. These results provide some support for the incremental clinical utility of FA, specifically in regard to social impairment, and suggest that individuals seeking treatment for FA may experience significant impairments in their social and interpersonal lives. These results are consistent with qualitative research [32], wherein people with FA described significant interpersonal difficulties as a result of FA. They reported that the shame, secrecy, and judgment by others regarding their difficulties with FA led them to withdraw from social situations, resulting in social and interpersonal harm.

Taken together, the results of the present research hold some implications for the clinical utility (i.e., prognosis, treatment plans, potential outcomes) of FA. Given the cross-sectional nature of our data, it would be premature to draw definitive conclusions regarding the prognosis or treatment of FA. Nonetheless, our findings identified factors worthy of further study using longitudinal and experimental designs, to examine whether they play a causal role in the generation or maintenance of FA symptoms or in recovery from FA. For example, given the significant association between FA and anxiety, it is plausible that excessive food consumption may represent a maladaptive coping mechanism to manage symptoms of anxiety, as was described by participants in an earlier qualitative study conducted by our team [32]. Using addictive behaviors to regulate one’s emotions is a hallmark characteristic of addictions. Previous reports have distinguished two types of emotion regulation goals that may motivate addictive behavior: reducing aversive emotional states such as anxiety (a form of negative reinforcement) or increasing positive affect via the introduction of a pleasurable stimulus (a form of positive reinforcement). Some addictive behaviors such as alcohol abuse may be more motivated by the former goal, whereas others, such as gambling, may be motivated by the latter [33,34]. Our findings suggest that, in FA, eating may be used primarily as a means of reducing aversive emotional states, rather than for increasing positive affect. This finding is consistent with a prior qualitative study where participants emphasized eating as a means of temporarily reducing aversive emotional states [32].

From a potential treatment perspective, if addictive-like eating represents a maladaptive response to anxiety, then clinicians may wish to employ an integrated treatment model to address difficulties with anxiety and FA simultaneously, by the same team of clinicians. Indeed, an integrative approach has been associated with improved outcomes compared to a sequential or parallel treatment approach when addressing multiple concerns [35]. Regarding social impairments, should social functioning be identified as a causal or perpetuating factor in the development and maintenance of FA symptoms, incorporating aspects of social skill training into the treatment plan may lead to improved prognosis, and social impairment may represent an outcome to track in progress monitoring. That said, more research is needed regarding the associations between FA and anxiety as well as social impairments before recommending potential treatment strategies for FA. Lastly, the results suggest that it may be helpful to monitor treatment outcomes not only for FA but also for related conditions given the high rates of psychiatric conditions, in particular anxiety disorders, and the strong associations between FA, binge eating, and depression.

### Limitations

Several limitations are worth noting. First, the cross-sectional design does not allow for causal inferences between FA and our variables of interest. Second, given the relatively small sample size, future research with larger samples is needed to replicate the findings of the present research. Having said that, the sample in the present study is one of the largest clinical samples of FA, and to our knowledge, the only study investigating clinical characteristics of people seeking treatment whose primary concern is FA. Third, the version of the MINI used in the present research captured DSM-IV diagnoses and as such did not capture binge eating disorder. Rather, in the present research, the well-known and used Binge Eating Scale was used to examine the relationship between FA and binge eating disorder. However, the BES was developed prior to the inclusion of binge eating disorder in the DSM-5 and thus was not designed to diagnose binge eating disorder [27]. Importantly, FA predicted social impairments when controlling for binge eating symptoms, providing some support for the clinical utility of FA. Lastly, although FA predicted social impairment when controlling for demographic factors and Body mass index, it is possible that other variables may have influenced the results. As such, future research controlling for other confounding variables would provide further support for our results.

## 5. Conclusions

In the past decade, there has been an increased empirical interest in FA. Although our knowledge of FA has grown exponentially, less is known regarding the clinical utility of FA, which impedes progress in developing efficacious treatments for this relatively common concern. The current study suggests that people seeking treatment for FA are likely to present with co-occurring psychiatric conditions and experience impairments in social functioning, over and beyond other psychopathology. Taken together, the results suggest FA holds some clinical utility. The results of the present research, as well as future research investigating the clinical utility of FA, may help in providing knowledge on the potential treatment possibilities for people with FA.

## Figures and Tables

**Table 1:** Frequencies of current psychiatric conditions met by participants using the Mini International Neuropsychiatric Interview (MINI).

Psychiatric Diagnoses	Yes	No
Major Depressive Episode	20 (47.6%)	22 (52.4%)
Dysthymia	4 (9.5%)	38 (90.5%)
Suicidality	11 (26.8%)	30 (73.2%)
Panic Disorder	4 (9.5%)	38 (90.5%)
Agoraphobia	9 (21.4%)	33 (78.6%)
Social Anxiety	8 (19.0%)	34 (81.0%)
Generalized Anxiety	26 (61.9%)	15 (38.1%)
Obsessive Compulsive Disorder	6 (14.3%)	36 (85.7%)
Post-Traumatic Stress Disorder	1 (2.4%)	41 (97.6%)
Alcohol Abuse/Dependence	1 (2.4%)	41 (97.6%)
Substance Abuse/Dependence	0 (0.0%)	42 (100.0%)
Anorexia Nervosa	0 (0.0%)	42 (100.0%)
Anorexia Nervosa Binge/Purge Type	0 (0.0%)	42 (100.0%)
Bulimia Nervosa	7 (16.7%)	35 (83.3%)
Any Current Condition	35 (83.3%)	7 (16.7%)

**Table 2:** Means, standard deviation, and correlations between our variables of interest.

	M	SD	1	2	3	4	5	6	7	8	9
1. YFAS2.0	142.45	34.89	-	0.42 **	0.26	0.28	0.50 ***	−0.37 *	−0.32 *	−0.44 **	−0.29
2. BES	30.46	7.60		-	0.63 ***	0.43 **	0.36 *	−0.54 **	−0.63 ***	−0.12	−0.22
3. FCQ-T	166.39	30.27			-	0.22	0.30 *	−0.35 *	−0.39 **	−0.09	−0.14
4. BDI	20.31	10.37				-	0.53 **	−0.86 ***	−0.73 ***	−0.43 **	−0.53 ***
5. BAI	16.66	10.42					-	−0.57 ***	−0.55 ***	−0.25	−0.42 **
6. WHO_Psyc	10.77	2.94						-	0.81 ***	0.52 ***	0.64 ***
7. WHO_Phys	12.07	2.74							-	0.42 **	0.51 ***
8. WHO_Soc	11.68	3.63								-	0.56 ***
9. WHO_Env	12.65	2.36									-

Note. YFAS 2.0 = Yale Food Addiction Scale 2.0; BES = Binge Eating Scale; FCQ-T = Food Craving Questionnaire—Trait; BDI = Beck Depression Inventory; BAI = Beck Anxiety Inventory; WHO = WHOQOL-bref; Psyc = psychological; Phys = physical; Soc = social; Env = environmental. * *p* < 0.05; ** *p* < 0.01; *** *p* < 0.001. M = Mean, SD = Standard deviation.

**Table 3:** Multiple regression results predicting psychological impairments measured using the WHOQOL-bref.

Psychological Impairment	b	LL	UL	SE b	B	adj. R^2^	R^2^C
Model						0.78	0.81 ***
Constant	17.62 ***	14.92	20.33				
Food Addiction	−0.01	−0.02	0.01	0.01	−0.08		
Anxiety Symptoms	−0.01	−0.06	0.05	0.03	−0.03		
Depression Symptoms	−0.22 ***	−0.27	−0.17	0.03	−0.78 ***		
Binge Eating Symptoms	−0.07	−0.13	−0.01	0.04	−0.19		
Food Craving	0.003	−0.02	0.02	0.01	0.04		

Note. b = unstandardized beta, LL = Lower limit, UL = Upper limit, SE b = Standard error of unstandardized beta, B = standardized beta, adj.R^2^ = adjusted R^2^, R^2^C = R^2^ Change. *** *p* < 0.001.

**Table 4:** Multiple regression results predicting physical impairments measured using WHOQOL-bref.

Physical Impairment	b	LL	UL	SE b	B	adj. R^2^	R^2^C
Model						0.61	0.66 ***
Constant	19.24 ***	15.68	22.79				
Food Addiction	0.01	−0.01	0.03	0.01	0.09		
Anxiety Symptoms	−0.05	−0.12	0.02	0.03	−0.17		
Depression Symptoms	−0.13 ***	−0.20	−0.06	0.03	−0.48 ***		
Binge Eating Symptoms	−0.14 **	−0.23	−0.04	0.05	−0.38 **		
Food Craving	−0.003	−0.03	0.02	0.01	−0.04		

Note. b = unstandardized beta, LL = Lower limit, UL = Upper limit, SE b = Standard error of unstandardized beta, B = standardized beta, adj.R^2^ = adjusted R^2^, R^2^C = R^2^ Change. ** *p* < 0.01, *** *p* < 0.001.

**Table 5:** Multiple regression results predicting social impairments measured using WHOQOL-bref.

Social Impairment	b	LL	UL	SE b	B	adj. R^2^	R^2^C
Model						0.28	0.37 **
Constant	16.57 ***	10.38	22.77				
Food Addiction	−0.05 **	−0.08	−0.02	0.02	−0.49 **		
Anxiety Symptoms	0.05	−0.08	0.17	0.06	0.13		
Depression Symptoms	−0.18 **	−0.29	−0.06	0.06	−0.50 **		
Binge Eating Symptoms	0.09	−0.08	0.26	0.09	0.19		
Food Craving	0.01	−0.03	0.05	0.02	0.09		

Note. b = unstandardized beta, LL = Lower limit, UL = Upper limit, SE b = Standard error of unstandardized beta, B = standardized beta, adj.R^2^ = adjusted R^2^, R^2^C = R^2^ Change. ** *p* < 0.01; *** *p* < 0.001.

**Table 6:** Multiple regression results predicting environmental impairments measured using WHOQOL-bref.

Environmental Impairment	b	LL	UL	SE b	B	adj. R^2^	R^2^C
Model						0.21	0.31 *
Constant	14.51 ***	10.28	18.74				
Food Addiction	−0.01	−0.03	0.01	0.01	−0.16		
Anxiety Symptoms	−0.02	−0.10	0.06	0.04	−0.09		
Depression Symptoms	−0.11 **	−0.19	−0.03	0.04	−0.48 **		
Binge Eating Symptoms	−0.001	−0.12	0.12	0.06	−0.004		
Food Craving	0.01	−0.02	0.04	0.02	0.15		

Note. b = unstandardized beta, LL = Lower limit, UL = Upper limit, SE b = Standard error of unstandardized beta, B = standardized beta, adj.R^2^ = adjusted R^2^, R^2^C = R^2^ Change. * *p* < 0.05; ** *p* < 0.01; *** *p* < 0.001.
